# Notes and News

**Published:** 1902-06-21

**Authors:** 


### NOTES AND NEWS.

The Royal Dental Hospital of London, Leicester Square, has received the sum of £500 from the trustees of Smith's (Kensington Estate) Charity.

One portion of the memorial in Newcastle-on-Tyne to the late Lord Armstrong is to be the completion of the buildings of the Durham University College of Science in Newcastle. The fund now amounts to £25,774.

The Mazawattee Tea Company have issued an illustrated edition of "God Save the King," the words and music of which are made to run through a series of elaborate designs in many colours, bringing in Royalties, flags, soldiers, sailors, and all sorts of matters appropriate to Coronation times in a highly ingenious manner.

Before the Royal Commission upon Alien Immigration Mr. W. Belcher, a councillor of the Borough of Stepney, gave evidence of numerous instances of overcrowding by foreigners in his borough. In one case he found 21 women working in a kitchen of 16 feet by 12 feet and 7 feet high, beds being all round the room, the food being black bread and coffee, and their pay 8d. or 9d. a week. In another case three beds were found in a cellar with 11 people sleeping in them, there being no ventilation whatever, only a small back window which was fixed and could not be opened.

The President of the Local Government Board has introduced a Bill to empower County Councils and other local authorities to make contributions to hospitals. These contributions may be either in the form of donations or subscriptions. The hospitals to which they may be made are defined as any hospital or infirmary or other institution for the care and treatment of persons suffering from any bodily injury, infirmity, or disease, or any mental infirmity, not carried on for purposes of private profit or gain, and supported wholly or in part by endowments or voluntary contributions.

The County Council of Pembrokeshire has recently paid the cost of sending patients bitten by rabid dogs to the Pasteur Institute in Paris. The Local Government Board has sanctioned payments by the Council in such cases.

Earl Roberts has consented to preside at the annual distribution of prizes to successful students of St. George's Hospital Medical School next October. Many George's men were out in South Africa under Lord Roberts, and a good muster is anticipated.

All the medical societies of Moscow are now under the official control of the Minister of Education. A council has been formed in direct connection with the University of Moscow, and upon it two members of each component society are elected to serve for three years.

Many hard things have been said about the Royal Army Medical Corps, but nothing that has ever been said about the personnel of that corps even approaches the stringent criticisms passed on the officers of the combatant branches of the army by the military education committee.

At the annual meeting of the Society for Training Teachers of the Deaf Dr. Warner, speaking of the difficulties which lay in their way from the lack of trained teachers, said that there was an excellent opening for women in the teaching of the deaf on the pure oral system.

The sums received since June 1st for the Coronation Gift to the Queen, at the offices, 72 Cheapside, E.C., include the following:—Messrs. Fremlin Bros., £26 5s.; the Countess of Dysart, £10 10s.; Major and Mrs. Hickman Morgan, £10; the Lady Mary Cooke, £5 5s.; Amy Lady Tate, £5 5s.; Miss Lyall, £5 5s.; Mr. and Mrs. W. C. Parsons, £5 5s. each; Mr. W. Melville Wills, £5 5s.; Miss Wood, £5 5s.; Mrs. Crossley, £5 5s.

At last Saturday's meeting of the managers of the Metropolitan Asylums Board, consideration was given to the question of hospital provision for fever and diphtheria patients, the clerk to the Board suggesting that the managers should be in a position to deal with 20,000 cases of scarlet fever a year. The Hospitals Committee, reporting upon the clerk's memorandum, recommended (*a*) "That in the opinion of the managers additional accommodation is required for the isolation of fever and diphtheria patients," and (*b*) "That it be referred to the Hospitals Committee to make inquiries with a view to the acquisition of more land for hospital purposes and to submit a scheme to the Board for their consideration." This recommendation, however, was strongly opposed, in view of the large increase which would shortly be effected in the number of beds provided for small-pox patients. Professor Smith, in moving that the matter be referred back to the committee for consideration and report, said the managers retained patients in their hospitals longer than was done in any other part of the kingdom. The Rev. G. W. Pope seconded the amendment, which, after discussion, was adopted.

